# Microbial biosurfactants: a review of recent environmental applications

**DOI:** 10.1080/21655979.2022.2074621

**Published:** 2022-06-06

**Authors:** Estefanía Eras-Muñoz, Abel Farré, Antoni Sánchez, Xavier Font, Teresa Gea

**Affiliations:** Composting Research Group (GICOM), Department of Chemical, Biological and Environmental Engineering, Escola d’Enginyeria, Universitat Autònoma de Barcelona, Cerdanyola del Vallès, Spain

**Keywords:** Bioremediation, biosurfactant, environmental applications, heavy metals, hydrocarbons, soil

## Abstract

Microbial biosurfactants are low-molecular-weight surface-active compounds of high industrial interest owing to their chemical properties and stability under several environmental conditions. The chemistry of a biosurfactant and its production cost are defined by the selection of the producer microorganism, type of substrate, and purification strategy. Recently, biosurfactants have been applied to solve or contribute to solving some environmental problems, with this being their main field of application. The most referenced studies are based on the bioremediation of contaminated soils with recalcitrant pollutants, such as hydrocarbons or heavy metals. In the case of heavy metals, biosurfactants function as chelating agents owing to their binding capacity. However, the mechanism by which biosurfactants typically act in an environmental field is focused on their ability to reduce the surface tension, thus facilitating the emulsification and solubilization of certain pollutants (*in-situ* biostimulation and/or bioaugmentation). Moreover, despite the low toxicity of biosurfactants, they can also act as biocidal agents at certain doses, mainly at higher concentrations than their critical micellar concentration. More recently, biosurfactant production using alternative substrates, such as several types of organic waste and solid-state fermentation, has increased its applicability and research interest in a circular economy context. In this review, the most recent research publications on the use of biosurfactants in environmental applications as an alternative to conventional chemical surfactants are summarized and analyzed. Novel strategies using biosurfactants as agricultural and biocidal agents are also presented in this paper.

## Graphical Abstract

**Figure:**
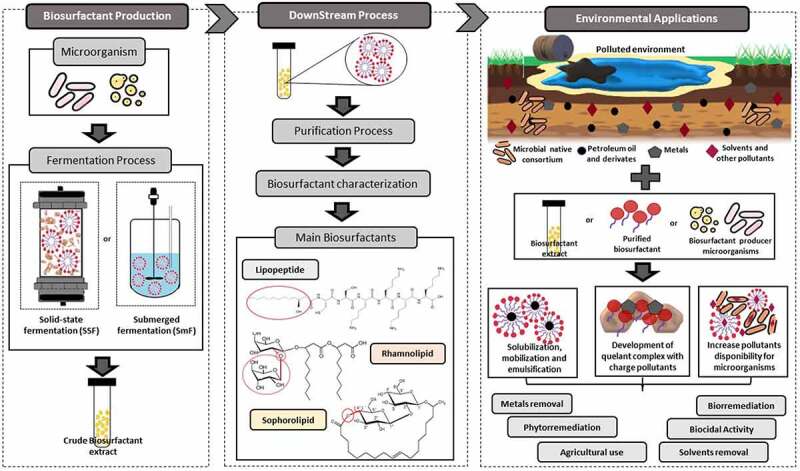

## Highlights

- Biosurfactants as substitutes for chemical surfactants for environmental application
- Rhamnolipids, sophorolipids and lipopeptides biosurfactants for bioremediation
- Biosurfactants can be applied by bioaugmentation and/or biostimulation strategy
- Nutrients and low molecular weight organic compounds promote bioavailability
- Biosurfactants enhance soil fertility, improve plant growth and reduce metal toxicity

## 1. Introduction

The exploitation of natural resources and increased industrial activities have resulted in serious environmental problems. Consequently, more effort is needed to solve environmental issues [1]. The most widely used energy resource is petroleum and its related products, whose transport and use often cause different forms of pollution, such as an increase in the biological and chemical oxygen demands in some soil or underground water environments [2]. Examples of this pollution are the spills of oil tanks in several parts of the world that have damaged millions of square kilometers of environmentally protected areas [3,4].

The metabolic pathways of some microorganisms use these petroleum-derived compounds. However, microorganisms cannot always degrade them completely; therefore, they can persist in soil and seawater for years. Owing to their low biodegradability, these pollutants represent a risk factor for native ecosystem organisms and their diversity [5]. One alternative is using chemical remediation processes using chelating agents and different chemical surfactants, such as sodium lauryl sulfate or acetyl trimethyl ammonium bromide, as complexing [6,7]. However, despite their effectiveness, these compounds are not biodegradable and can produce secondary pollutants [8].

**Figure 1.**
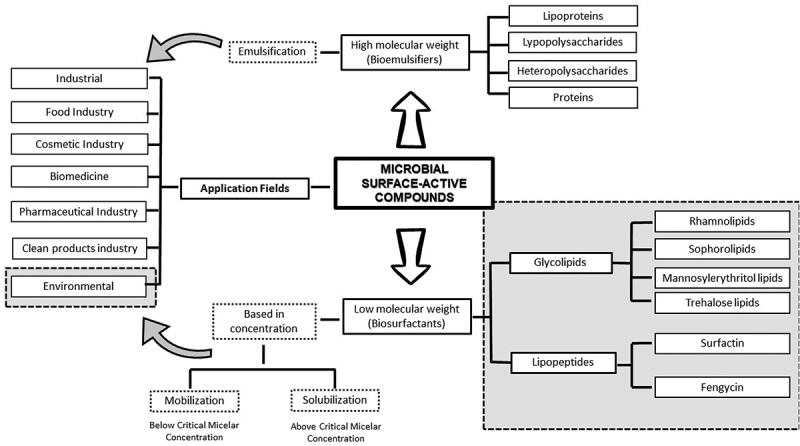
Microbial surface-active compound types and characteristics.

Consequently, it is necessary to search for natural substitutes for chemical surfactants and their use in an eco-friendly manner. Thus, microbial surface-active compounds (MSAC) have emerged as potential alternatives. These microorganisms produce compounds that are capable of solubilizing hydrophobic substrates such as hydrocarbons, lipids, oils, and antibiotics, allowing their use as carbon sources [9,10]. The capacity of MSAC to act as a foam producer, emulsifier, and solubilizing agent allows the solubilization, dispersion, and desorption of priority environmental pollutants such as petroleum derivatives, polycyclic aromatic hydrocarbons (PAHs), and heavy metals [11–13]. As shown in Figure 1, MSAC can be classified by its chemical properties and molecular weight as bioemulsifiers (high molecular weight) and biosurfactants (low molecular weight) [14]. Biosurfactants are the main group of MSAC that are used in environmental applications. They are secondary metabolites that can be produced from renewable energy sources by different types of bacteria, yeast, fungi, and archaea with antibacterial, antifungal, and antiviral properties [15,16]. They have low toxicity, high biodegradability, and are functional over a wide range of temperatures, pH, and salinity [7,17].

Biosurfactants are potential candidates for bioremediation because they increase the solubility and bioavailability of hydrophobic pollutants [4]. For other pollutants such as heavy metals, biosurfactants have been successfully used as complexing agents that can form micelles attached to the metal ions inside them [18]. In addition, biosurfactant-binding activity and their wide spectrum of metal selectivity promote the mobility and recovery of these pollutants [19,20].

A novel biosurfactant can be used as a biocide against pathogenic microorganisms, without the use of aggressive chemical methods. Furthermore, pathogenic microorganisms can develop resistance to chemical compounds such as atrazine [21]. [22] reported that biosurfactants could be an alternative to this problem because they present a very low critical micelle concentration (CMC), which is the minimum concentration required to reduce the surface tension to the maximum extent, a significant parameter to be considered in bioremediation processes. Compared with chemical surfactants, low concentrations of biosurfactants can be effective against pollutants without negative consequences [23,24].

While many reviews in the environmental field prioritize a specific biosurfactant and/or pollutant, this review focuses on a global overview of different environmental applications based mainly on bioaugmentation and biostimulation strategies. In response to the growing interest in this field, the main purpose of this critical review is to highlight the advantages of biosurfactants as alternatives to conventional chemical surfactants. A literature review was conducted over the last five years and 108 articles were analyzed. Some of which are summarized in the following sections according to each application case showing that apart from the typical use of biosurfactants in soil bioremediation, other emerging strategies are also being tested. The most recent approach involves the use of biosurfactants for agricultural purposes. The application of biosurfactants in wastewater treatment was not considered in this review, as recently reviewed by [23].

## 2. Microbial surface-active compounds origin and structure

Microbial surface-active compounds (MSAC) are amphiphilic molecules that contain hydrophobic and hydrophilic parts that promote the presence of interfaces between fluids with different polarities. The hydrophilic part generally consists of one of the following structures: amino acids, anionic or cationic peptides, and carbohydrates. The hydrophobic tail is generally composed of peptides, proteins, or fatty acids that can be saturated or unsaturated [8,18]. The capacity of MSAC to reduce the interphase tension varies according to its chemical structure. MSAC can be classified into two principal groups based on their molecular weight: low molecular weight (glycolipids and lipopeptides) and high molecular weight (polysaccharides, lipopolysaccharides, proteins, and lipoproteins). Low-molecular-weight biosurfactants are more effective in reducing surface tension, while high-molecular-weight biosurfactants are better for stabilizing oil-water emulsions [14,17,25,26]. The chemical differences in the molecular structure of different biosurfactants are directly related to their biological activities and applications [27].

Low-molecular-weight biosurfactants are most commonly used in the environmental field. Glycolipids, which are composed of mono-, di-, tri-, and tetra-saccharides in combination with one or more aliphatic or hydroxyaliphatic acid chains, are classified as trehalolipids, mannosylerythritol lipids (MELs), rhamnolipids, and sophorolipids [23]. Trehalolipids are composed of trehalose disaccharides. Each glucose is linked to a fatty acid (mainly mycolic acids) by an α-1-glycosidic bond, and its production is associated with species of *Mycobacterium, Corynebacterium*, and *Nocardia* [28,29]. MELs are produced by Microorganisms of the genera *Pseudozyma* and *Ustilaginaceae* [30].

Rhamnolipids are mainly produced by *Pseudomonas aeruginosa* and were the first biosurfactants described [31]. They are formed by mono- and disaccharides of rhamnose linked by a glycolic bond to a β-hydroxy fatty acid molecule. They are widely used in oil recovery processes and are used in the agricultural sector to control plant pathogens and improve soil quality [32,33]. Sophorolipids are extracellular biosurfactants that were first reported in the early 1960s as the main producer microorganisms *Starmerella bombicola* strains [25,34]. Their molecular structure can be cyclic or acyclic, with a variation in the sophorolipid sugar (lactonisation or acetylation) and fatty acid length (16 or 18 carbon atoms) with different degrees of saturation. Various studies have shown that sophorolipids can be produced from water-insoluble substrates (organic waste), making them attractive for industrial applications based on a circular economy strategy [35–37].

Among lipopeptides, cyclic lipopeptides such as gramicidin S and polymyxin have antibacterial activity because of their ability to solubilize membrane enzymes. Surfactin produced by *Bacillus subtilis* also belongs to this group and is considered the most effective biosurfactant for reducing surface tension owing to its low CMC [38. According to [13], surfactin is pH-regulated beyond pH 7.4, which allows its reuse (over 100 times) and improves the demulsification efficiency as its concentration increases. Lipopeptide production, properties, types, and applications have also been reviewed [14].

According to the literature, sophorolipids, rhamnolipids, and lipopeptides have potential applications [8,39,40]. Therefore, several companies have announced their interest in the use of biosurfactants in their products. Consequently, some companies have increased their production: rhamnolipids from AGAE Technologies Company (USA), GlycoSurf (USA), and Jeneil Biosurfactant Co. LLC (USA), and sophorolipids from Synthezyme LLC (USA) and Ecover Eco-Surfactant (Belgium). Both compounds are pioneers of their use in the environmental field [41–43].

## 3. Production of biosurfactants

### 3.1. Microorganisms and growing media

**Figure 2.**
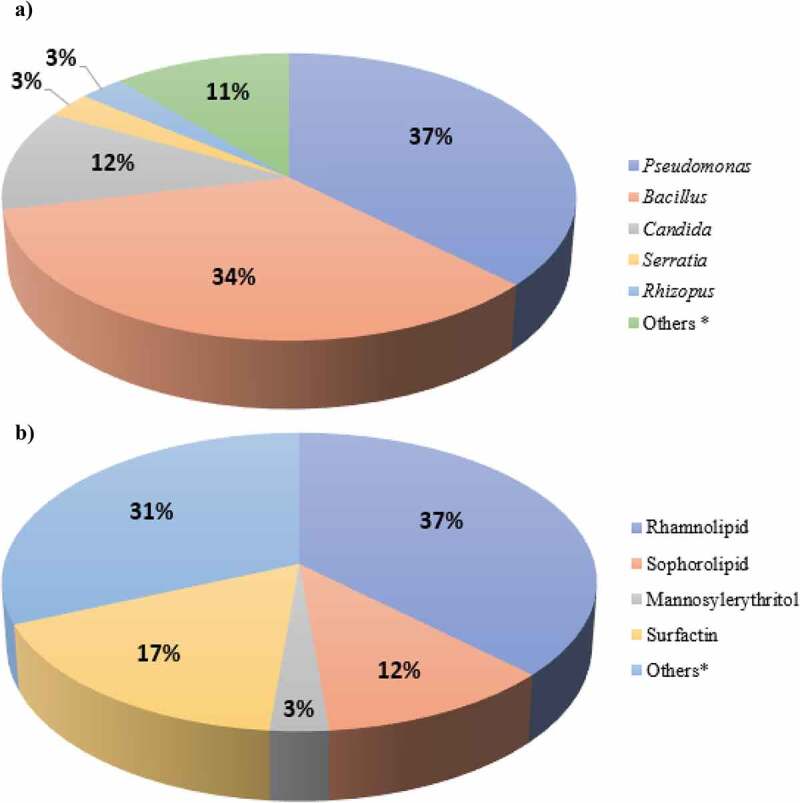
Main microorganisms and biosurfactants produced: a) Genera of biosurfactant producer microorganisms, *Others group also involve a combination of genera; b) Main biosurfactants produced according to literature, *Others group also involve a mix of biosurfactant or unknown cases.

According to the literature, different types of microorganisms belonging to the genera *Pseudomonas, Bacillus, Candida, Rhodococcus* and *Corynebacterium* are used to produce biosurfactants, with *P. aeruginosa* being the most commonly used for rhamnolipid production, according to the reviewed cases carried out between 2015 and 2021 for the production section (n = 36) (Figure 2) [44]. Because of the high market demand for biosurfactants, biotechnological tools are being used to identify biosurfactant production pathways and to obtain hyperproducing strains or recombinant mutants. For example, a novel *S. bombicola* mutant (ΔatΔsble) produces various sophorolipids called bolaforms [45]. This molecule structure consists of two sophorose units on each side of the hydrophobic tail. It is more stable at higher pH conditions, which impedes its application in a wide range of sectors, such as biomedicine for drug delivery [46].

To produce biosurfactants, microorganisms can use various compounds as carbon sources for their growth. The most common carbon sources are glucose and glycerol (Table 1). Because glucose is obtained from food sources and is widely used as an industrial feedstock, it increases biosurfactant production costs [47]. As an alternative, [48] reported a detailed review of feedstocks used for biosurfactant production. When complex substrates are used, impurities can be found in the final fermented extract. For example, as [49] described, fermentation using kerosene to produce a non-cytotoxic biosurfactant with *Serratia* sp. results in a high amount of impurities in the downstream phase.

**Table 1.** Examples of biosurfactants used in environmental applications and fermentation parameters of their production

		Operating Conditions								
Biosurfactant	Producer Microorganism	Carbon Source	Medium and Supplement	Volume (mL)	T (°C)	pH	Time (h)	Speed (rpm)	Yield (g L^−1^)	Reference
***Glycolipids***										
Rhamnolipid	*Pseudomonas aeruginosa* TGC01	Glycerol 4% (w/v)	Mineral salt medium and (g L^−1^): 4.00 NaNO_3_	500	30	-	96	150	11.00	105
Rhamnolipid	*Lysinibacillus sphaericus* IITR51	Glycerol 1.5% (w/v)	Basal salt medium	8	30	7	72	160	1.60	35
Rhamnolipid	*Pseudomonas aeruginosa* DR1	Mango oil and glucose 1% (w/v) Mango oil 1% (w/v)	Mineral salt medium	250	30	-	96	-	2.80 1.80	98
Rhamnolipid	*Pseudomonas aeruginosa*	Glucose 6% (w/v)	Mineral salt medium	250	37	-	168	330	3.80	56
Rhamnolipid	*Pseudomonas aeruginosa* SR17	Glucose 2% (w/v) and paneer whey waste Crude oil 2% (w/v)	Mineral salt medium	500	35	7	48	150	4.80 2.70	33
Rhamnolipid	*Pseudomonas aeruginosa*	Glycerol, glucose, mannitol, molasses and n-hexadecane at 2% (w/v)	Mineral salt medium	-	35	-	120	150	0.84	73
Rhamnolipid	*Pseudomonas aeruginosa* PBS	Glucose 5% (w/v) and kerosene 3% (w/v)	(g L^−1^): 5.00 KNO_3_, 1.00 KH_2_PO_4_.2H_2_O 1.00 K_2_HPO_4_.2H_2_O 0.20 MgSO_4_·7H_2_O 0.02 CaCl_2_·2H_2_O	100	37	7	72	150	2.65	106
Rhamnolipid	*Pseudomonas aeruginosa* PA1	Glycerol 2% (w/v)	Nutrient Broth	-	28	-	288	180	-	22
Rhamnolipid	*Pseudomonas* LSH-7	Crude oil 0.5% (w/v)	Mineral salt medium and (g L^−1^): 3.00 (NH_4_)_2_SO_4_ 2.00 K_2_HPO_4_	250	25	7.5	168	120	-	75
Mono- and di- rhamnolipid	*Bacillus algicola* 003-Phe1, *Rhodococcus soli* 102-Na5, *Isoptericola chiayiensis* 103-Na4, and *Pseudoalteromonas agarivorans*SDRB-Py1	Mannitol 2%, glucose, glycerol, starch, and crude oil 1% (w/v)	Mineral salt medium and (g L^−1^): 10.00 (NH_4_)_2_SO_4_ 0.50 yeast extract	100	28	7	72 168	180	2.00 strain 003-Phe1 and 1.20 SDRB-Py1	88
Rhamnolipid	*Pseudomonas aeruginosa*	Glycerol 2% (v/v)	Mineral salt medium	250	30	7	192	180	5.07	6
Rhamnolipid	*Pseudomonas aeruginosa* UCP 0992	Corn steep liquor 0.5% and vegetable oil residue 4% (v/v)	-	1 200	28	7	120	225	26.00	52
Rhamnolipid	*Pseudomonas aeruginosa*	Glucose 1% (w/v)	Mineral salt medium	250	30	7	168	180	1.80	87
Sophorolipid	*Candida albicans* and *Candida glabrata*	Glucose 2% (w/v)	Synthetic defined medium with yeast nitrogen base 0.67%	200	30	-	72	150	1.32 *C. albicans* and 1.60 *C. Glabrata*	102
Sophorolipid	*Starmerella bombicola* ATCC 22214	Winterization oil cake (WOC) and molasses	-	500 22 000 100 000	30	5.6	168	-	0.16 0.19 0.14 (gSL gDM^−1^)	37
Sophorolipid	*Candida sphaerica* UCP 0995	Corn steep liquor 9% and ground-nut oil refinery residue 9% (v/v)	Basal medium	250 50 000	27	-	144	150	8.00 21.00	36
Sophorolipid	*Candida tropicalis*	Corn liqueur 4%, molasses 2.5% and canola frying oil 2.5% (v/v)	-	500 3 00050 000	28	6	144	200	9.50 20.00 27.00	5
MELs	*Pseudozyma* sp. NII 08165	Soybean oil, diesel, kerosene, and petroleum	Bushnell Hass broth	-	30	7	216	200	-	24
***Lipopeptide***										
Surfactin	*Bacillus velezensis* MHNK1	Residual frying oil 2% (v/v)	Mineral salt medium	500	37	-	48	150	0.83	21
Surfactin	*Bacillus subtilis* ATCC 21332	Glucose 4% (w/v)	Mineral salt medium	250	30	7	72	200	-	13
Surfactin	*Bacillus methylotrophicus*	Soybean oil 2% (v/v) and whey	(g L ^−1^):1.00 (NH₄)₂SO₄ 0.03 NaBr1.00 CuSO_4_.5H_2_O 0.81 MgSO_4_.7H_2_O 0.31 ZnSO_4_.7H_2_O	250	30	6.7	120	200	-	69
Surfactin	*Bacillus amyloliquefaciens* SAS-1 and *Bacillus subtilis* BR-15	Glycerol 5% (w/v) and glucose 2.17% (w/v)	Mineral salt medium with yeast extract 0.5% (w/v)	-	37.5	7	72	-	-	62
Surfactin and others	*Bacillus lichenimorfis* L20	Glucose, sucrose and lactose 10 g L^−1^	Mineral salt medium	3 000	37	-	168	120	-	107
Cyclic lipopeptide	*Bacillus tequilensis*	-	Mineral salt medium	-	35	-	168	150	-	97
Crude lipopeptide	*Bacillus amyloliquefaciens*An6	Glucose20 g L^−1^	Landy medium	100	30	7	72	160	-	108
Crude lipopeptide	*Bacillus subtilis* CN2	Glycerol 4% (v/v)	Mineral salt medium	500	37	7	96	150	-	109
Crude lipopeptide	*Paenibacillus dendritiformis*	Sunflower oil 3% (v/v) and anthracene 0.01% (w/v)	Mineral salt medium	1 000	37	-	120	150	6.00	81
Crude lipopeptide	*Bacillus subtilis* AS2,*Bacillus licheniformis* AS3and *Bacillus velezensis* AS4	Crude oil2% (w/v)	Zobell marine medium	-	37	7	120	150	-	71
Crude lipopeptide	*Bacillus cereus*UCP 1615	Waste frying soybean oil 2% (w/v)	Mineral salt medium with:(g L^−1^): 1.00 yeast extract 1.20 peptone	250 1 200 3 000 50 000	28	7	48	250	3.504.304.604.11	54
Crude lipopeptide	*Bacillus subtilis*	Glucose 15 g L^−1^and crudeoil 1% (w/v)	(g L^−1^): 0.40 MgSO_4_ 1.07 NH_4_Cl 1.49 KCl 18.90 Tris–HCl 10.00 peptone	-	30	-	72	160	-	3
Crude lipopeptide	*Pseudomonas aeruginosa*	Glycerol 60 g L^−1^	Mineral salt medium	1 000	37	7	144	150	9.80	74
Syringafactin	*Pseudomonas putida*	Glucose2.5 g L^−1^	Tryptic soy broth	100	30	-	48	160	-	64
***Other biosurfactants***										
Biosurfactant extract	*Serratia marcescens andSerratia nematodifila*	Kerosene 2% (v/v)	Bushnell Haas broth	-	37	7	120	135	-	49
Biosurfactant extract	*Enterobacteriaceae, Pseudomonas, Microbacterium* and *Rhodanobacteraceae*	Colza oil and glucose 20 g L^−1^	(g L^−1^):0.10 NH_4_NO_3_0.25 K_2_HPO_4_ 0.25 Na_2_HPO_4_ 0.25 NaCl	100	20	-	168	180	-	53
Biosurfactant extract	*Rhizopus arrhizus*UCP1607	Crude glycerol and corn steep liquor	(g L^−1^):0.20 KH_2_PO_4_0.20 MgSO_4_·7H_2_O	100	28	5.5	96	150	1.74	110
Biosurfactant extract	*Wickerhamomyces anomalus*CCMA 0358	Glucose 2 g L^−1^and olive oil20 g L^−1^	(g L^−1^):4.00 yeast extract4.00 (NH_4_)_2_SO_4_	500	28	-	24	200	-	55

Occasionally, biosurfactant production media must be supplemented with yeast extract or macronutrients in the form of chlorine, sulfate, or phosphate salts of different metals (Na, Mg, K, Ca, or Fe, among others). More recent evidence reports that the main fermentation media use the mineral fermentation medium (40% of the production reviewed cases), which may slightly vary in composition for each biosurfactant production case (Table 1). Because the use of pure substrates and media supplementation has an economic impact, the use of low-cost byproducts and waste as feedstock for sophorolipid production has also been reviewed [25,34,50].

### 3.2. Fermentation process

Submerged fermentation (SmF) and solid-state fermentation (SSF) are the main bioprocesses used in biosurfactant production. SSF offers advantages such as avoiding substrate inhibition, allowing the use of industrial residues and agro-industrial waste, and low energy consumption, which make the processes rentable. However, compared with SmF, it presents drawbacks associated with substrate heterogeneity, operational monitoring, and difficult downstream processing [32,51].

Most biosurfactants have reported production volumes of 100–1200 mL at the laboratory scale. Few papers report higher volumes, which are from bench scale (around 3 L), and very few cases present an SmF scale-up process at the pilot scale of 20 L and 50 L with production yields between 0.83 g L^−1^ and 27 g L^−1^. The highest SmF yield at the lab scale was obtained at a volume of 1.2 L by *P. aeruginosa* UCP 0992 (26 g L^−1^) to obtain a rhamnolipid using corn steep liquor and vegetable oil residue as fermentation substrates [52]. The lowest yield at the same production scale was 500 mL (0.83 g L^−1^) for surfactin production using *B. velezensis* MHNK1 [21]. At production volumes of 50 L, better sophorolipid yields (21 g L^−1^ and 27 g L^−1^) were found using *Candida* species and high-fat content residues [5,36]. However, biosurfactant purity and efficiency should be considered in process evaluation because this comparison was made only according to the production scale. According to previous studies, highly productive strains, optimized fermentation conditions, and the use of cheaper substrates are essential for affordable costs and expanding the industrial production and environmental applications of biosurfactants [52–54].

For this reason, emerging alternatives, such as low-cost substrates (organic waste and side streams) and solid-state fermentation (SSF), are being studied to enhance the process [25]. 36,reported a biosurfactant scale-up process with *Candida sphaerica UCP 0995* for application in seawater contaminated with motor oil. The biosurfactant extract with a capacity of dispersing 90% of oil drops in seawater was produced using two low-cost substrates (corn steep liquor and ground-nut oil refinery residue) in 20-L bioreactors obtaining a yield of 21 g L^−1^ at 144 h, similar to that obtained at lab-scale. Comparably, 37,used winterization oil cake and molasses as low-cost substrates for biosurfactant production in an SSF process with 22-L and 100-L reactors with *S. bombicola*, achieving a yield of 0.19 gSL gDM^−1^ and 0.14 gSL gDM^−1^, respectively, showing the production adaptability that this microorganism presents. It should be highlighted that SSF is carried out using solid substrates where microorganisms grow in the absence of free water. For this reason, yields were expressed as grams of biosurfactant per gram of initial dry matter in the solid substrate [51].

The fermentation conditions depend on the microorganisms and biosurfactant to be produced (Table 1). The fermentation temperature reported for different microorganisms is in the range of 25–40°C, with 30°C being the most frequently used. At 30°C, microorganisms belonging to the genera *Pseudomonas* and *Bacillus* are the most studied. The fermentation time oscillated in a very wide range, from 48 h to 288 h. [55] described that in comparison with other yeast strains, *Wickerhamomyces anomalus* CCMA 0358 is a faster biosurfactant producer with a fermentation time of 24 h, contributing to the reduction of production costs. Agitation was mainly in the speed range of 135–250 rpm, although higher values were found [56]. Although high-speed agitation can reduce the cell count in yeast, it has been reported that in some cases, it increases biosurfactant production [55].

The recovery of the final product (downstream process) represents approximately 60% of the production costs and makes the process expensive in comparison with chemical surfactant production [55,57]. For submerged fermentation, centrifugation was performed before the downstream processes to remove biomass. Nevertheless, the downstream process is vaguely commented on in the review cases; a recent review of this topic was published by [58]. Techniques, such as precipitation, extraction, and crystallization, have been described for purification purposes. Precipitation is normally carried out under acidic conditions. Extraction is usually carried out with organic solvents such as mixtures of 2:1 (v/v) methanol:chloroform, 3:1:1 (v/v) methanol:n-hexane:water, or 1:4 (w/v) ethyl acetate:n-hexane [59,60]. In addition, extraction with solvents is the most commonly reported procedure for quantifying biosurfactants obtained by SSF. For crystallization, buffers such as phosphate and phthalate were added to obtain the purified biosurfactant. [56] used zero-valent iron nanoparticles for purification purposes. 61,described another novel method which uses integrated gravity separation for scaling up sophorolipid recovery.

Biosurfactant-specific properties are mainly characterized by collapse tests, oil drops, surface tension, emulsification index, foaming index, and CMC [23]. Previous studies have reported that a microorganism is considered a biosurfactant producer if it can reduce the surface tension to values under 40 mN m^−1^ [62]. The purification methods and structural characterization include thin-layer chromatography with silica gel plates (TLC), liquid chromatography coupled with mass spectrometry (LC-MS), and Fourier transform infrared spectrophotometry (FTIR) [63].

In conclusion, biosurfactant production efforts have focused on the use of new feedstock substrates, mainly industrial waste, and low-cost substrates, which can be combined with SSF to make the process rentable. Nevertheless, some authors have reported obtaining a crude biosurfactant extract owing to the high purification costs and poor downstream process information. Therefore, biosurfactant production should be complemented with an economic analysis in which the purification process and future applications of biosurfactants must be considered.

## 4. Environmental uses of biosurfactants

Biodegradation of hydrophobic organic contaminants is typically limited by their solubility. Therefore, the use of biosurfactants is a well-explored technique as they can mobilize, emulsify, and solubilize these compounds, thus improving biodegradation processes in soil bioremediation [64] and wastewater treatment [23]. Reducing the interfacial tension enhances the interaction between the water and oil–solid matrix and decreases the capillary forces that prevent the migration of the pollutant. The use of biosurfactants or biosurfactants-producing microorganisms, which are resistant to pollutants, can be applied in this field [65,66].

Biosurfactant environmental applications are based on two principal interaction mechanisms. On one hand, the presence of biosurfactants increases substrate bioavailability. On the other hand, it promotes interaction with the cell surface by increasing its hydrophobicity, allowing hydrophobic substrates to interact with bacterial cells [62,67,68]. The succeeding sections review the main environmental applications of biosurfactants reported in the literature.

### 4.1. Petroleum bioremediation

Approximately 60–90% of petroleum compounds are biodegradable, although the biodegradation process can be very slow. For this reason, biosurfactants are used to enhance pollutant bioavailability, which is an important factor for oil remediation [69]. In the reviewed literature, the material to clean is commonly soil from an oil exploitation area or artificially contaminated soil simulating the same conditions (Table 2). [44] confirmed that petroleum sludge or waste is a good substrate for producing biosurfactants using microorganisms isolated from hydrocarbon-contaminated sites.

**Table 2.** Summary of biosurfactant application for petroleum bioremediation

Reported experiment	Biosurfactant	Microorganism or consortium	Substrate	Biosurfactant efficiency (%)	Reference
***Biostimulation***					
Hydrocarbon degradation (TPH) an improved electroremediation with five different surfactants from an oil-contaminated soil	β-cyclodextrin	*Clostridium*, *Bacillus* and *Pseudomonas*	Soil contaminated with hydrocarbons from an oil field	TPH removal of: 77.00% with Lecithos, 56.00% with SDS and 50.00% with β-cyclodextrin	12
Application of different biosurfactant concentration to improve the biodegradation of hydrocarbon compounds (TPH)	Rhamnolipid	*Pseudomonas aeruginosa* SR17	Soil contaminated with crude oil	At 1.50 g L^−1^ biosurfactant TPH removal of: 86.10% for soil with 6800 rpm 80.50% for soil with 8500 rpm	73
Evaluation of crude oil remediation by bio-electrokinetic technique using biosurfactant to increase process efficiency	Lipopeptide	*Bacillus subtilis* AS2, *Bacillus licheniformis* AS3 and *Bacillus velezensis* AS4	Soil contaminated with crude oil	Crude oil removal of: 88.00% by strain AS2, 92.00% by strain AS3 and 97.00% by strain AS4	71
Stimulation of crude microbial bioremediation of offshore marine oil with different doses of chemical surfactants (GM-2 and DOSS) and biosurfactants	Rhamnolipid	*Pseudomonas* LSH-7’	Marine offshore oil spill sample	73.94% removal at 15.00% of crude oil.	75
Crude biodegradation effect of synthetic surfactants (Tween 80, Brij30, SDS and anionic synthetic surfactant) and biosurfactants on an indigenous microbial community	Cyclic lipopeptide	*Bacillus subtilis* WU-3	Water samples from an oil field	52.60% crude oil degradation at 0.1 CMC and 53.60% at 0.2 CMC biosurfactant concentration	74
Integrated application of biochar, biosurfactant and nitrogen fertilizer in the removal of polluting crude in coastal wetland	Rhamnolipid	Microbial community	Soil of a wetland artificially contaminated with crude	80.90% TPH reduction from the complex of biochar, nitrogen and biosurfactant	4
Evaluation of biosurfactants efficiency removing motor oil from laboratory sand samples and their comparison with synthetic surfactants	Biosurfactant extract	*Candida sphaerica* and *Bacillus* spp.	Sand packed column contaminated with motor oil	93.00% removal by *C.* *sphaerica* biosurfactant 43.00% removal by *Bacillus* sp biosurfactant	65
***Bioaugmentation***					
Application of isolated *Pseudomonas* biosurfactants producing strains (E311, E313, E39) and *Rhodococcus eritropolis* T902.1 as positive control for diesel biodegradation	Syringafactin	*Pseudomonas putida* and *Pseudomonas* spp.	Sand bioreactors contaminated with diesel	68.00% by strain E311, 57.00% by strain E313 and 55.00% by strain E39 TPH removal.	64
Isolation of hydrocarbon degrading microorganisms and germination experiments with kerosene, bacterial cultures and their produced biosurfactant	Biosurfactant extract	*Serratia* sp. KDS	Kerosene	87.54% for diesel and 85.48% for kerosene removal	49
***Complementary biostimulation and bioaugmentation***					
Diesel biodegradation by a consortium adding a biological emulsifier and/or co-inoculation with biosurfactant producer microorganisms	Lipopeptide	*Acinetobacter radioresistens* RI7 and *Bacillus subtilis* SPB1	Soils modified with hydrocarbons	32.67% diesel degradation	15
Petroleum oil degradation (TPH) using a contaminating soil isolated consortium adding biosurfactants to enhance the biodegradation process	Sophorolipid	*Enterococcus*, *Vagococcus*, *Sphingomonas* and *Proteus*.	Petroleum hydrocarbons in contaminated soils	44.50% by isolated consortium 57.70% by isolated consortium plus 1.50 gSL kg^−1^	79
Evaluation of the effects of a biosurfactant extract supplemented with the same producer microorganism for diesel oil removal	Biosurfactant extract	*Bacillus methylotrophicus*	Soil samples contaminated with 20% diesel oil	60.48% contaminant removal on biostimulation treatment and 57.92% on bioaugmentation treatment	69

70, reported that *Pseudomonas* species are the main biosurfactant producers in hydrocarbon-polluted environments. However, recent studies conducted by 71,found that marine microorganisms such as *B. subtilis* AS2, *Bacillus licheniformis* AS3, and *Bacillus velezensis* AS4 can also be found in this environment. They can produce biosurfactants with high emulsification values for low molecular weight hydrocarbons that also present crude oil degradation efficiencies of 88%, 92%, and 97%, respectively. As petroleum is a complex mixture, many authors have used the parameter of total petroleum hydrocarbon (TPH) reduction to report the efficiency of the biodegradation process to compare different studies.

#### 4.1.1. Biostimulation for petroleum bioremediation

Biostimulation is a strategy based on the addition of a commercial or self-produced biosurfactant (alternatively, a final fermentation extract) that can be applied alone or in combination with other compounds [72]. Biostimulation is one of the most used bioremediation strategies when applying biosurfactants, with biodegradation efficiencies ranging 50–97%.

Rhamnolipids and lipopeptides are the most commonly reported biosurfactants used in biostimulation. [4] applied rhamnolipids together with biochar and a nitrogen fertilizer to promote the activity of oil-degrading microorganisms and increase the fertility of saline mash soils. TPH removal was 19.7% using only rhamnolipid. A maximum TPH reduction of 80.9% was achieved with the integrated biochar–rhamnolipid–nitrogen fertilizer. Nutrient stimulation with nitrogen and phosphorus can affect the persistence of hydrocarbons. However, when compounds such as biochar are applied to retain them and biosurfactants are applied as solubilisers, they do not present this adverse effect. According to [73] contaminants like benz(d)anthracene, benz(b)fluorene and fluoranthene were eliminated by heterotrophic bacterial population with a TPH removal efficiency in the range of 80.5–86.1% using rhamnolipid biostimulation at 1.5 g L^−1^ concentration in a period of six months. This efficiency was higher than that obtained with the commercial surfactant SDS (sodium dodecyl-sulfate, anionic surfactant).

Most of the studied biostimulation cases compared biosurfactants with chemical surfactants as reference compounds used in oil bioremediation. [65] showed the highest effectiveness to remove motor oil from sand with *C. sphaerica* and *Bacillus* sp. biosurfactant crude extract (93% and 43%, respectively], in contrast with Tween 80 and Triton X-100 at different CMC concentrations (range of 40–80%). [62] applied different concentrations of a biosurfactant (0.05–2 g L^−1^) produced by *Bacillus amyloliquefaciens* An6 for diesel oil removal and presented a comparison with chemical surfactants, such as Tween 80 and SDS. The obtained results confirmed that the produced biosurfactant has high emulsifying effect and apparently promotes diesel solubilization at a concentration of 1 g L^−1^ with the highest efficiency of 71.54%. They also reported that the intrinsic degradation capacity of the microorganisms is stimulated by high concentrations of their own biosurfactant, owing to a pseudo-solubilization process that reduces the interfacial tension between the cell surface and the pollutant. This external addition of biosurfactants promotes the biodegradation of complex compounds and favors the process when microbial growth is slow.

[12] compared five different types of surfactants, including biosurfactants and cationic, anionic, nonionic, and ampholytic surfactants, to improve electroremediation treatment. Results showed a TPH degradation rate of 50% with the biosurfactant β-cyclodextrin, only surpassed by the ampholytic surfactant (77%) and the anionic surfactant (56%). However, these findings are in contrast with those of [74], who evaluated crude oil biodegradation in an autochthonous microbial community by the effect of four synthetic surfactants: SDS, sodium dodecyl benzene sulfonate (LAS), anionic surfactant (Brij 30, nonionic chemical surfactant), Tween 80, and a cyclic lipopeptide as a biosurfactant. The results showed that the highest degradation of crude oil (54.4%) was obtained with SDS at a concentration of one-fold CMC, and the rate decreased (51.1%) at a dose of two-fold CMC. In addition, Brij 30 had a negative effect in all the concentrations tested. Regarding the applied biosurfactant, biodegradation was improved at low concentration of 0.2 times that of CMC (53.6%). However, it presented an inhibitory effect at higher concentrations (equal to CMC, 41.3%) in comparison with the control without biosurfactant (50.5%). This may be because of their antibacterial activity and the possible cross-interaction between pollutants and surfactants, or biosurfactants that might interfere with the solubilization process [73,75].

According to these studies, biosurfactants are effective at low concentrations and show higher efficiency rates in biodegradation processes in adverse environments than chemical surfactants [76]. Biosurfactants can improve oil solubility and rapidly emulsify oil into tiny drops that can be biodegraded by microorganisms. However, an inhibitory effect was observed at high concentrations because of their antibacterial activity. For example, surfactin and rhamnolipid at concentrations of 80 mg L^−1^ and 240 mg L^−1^, respectively, have an inhibitory effect in the growth of hydrocarbon biodegradation strains that is attributed to their cell disruption capacity, which must be considered [24].

#### 4.1.2. Bioaugmentation for petroleum bioremediation

Bioaugmentation is based on the introduction of microorganisms into the soil, which enhances bioremediation [72]. In this case, the addition of biosurfactant- producing microorganisms to the contaminated systems increased hydrocarbon solubilization and bioavailability, obtaining biodegradation efficiencies between 32.67% and 87.54% (Table 2).

69, observed that bioaugmentation could generate a competition between native and introduced microorganisms. Their results showed a similar percentage of diesel oil removal in soil (57.92%) for bioaugmentation treatments and the control (approximately 58%). Other authors have highlighted that external microorganisms need adaptation time and often do not survive in highly contaminated environments. The alternative is bioaugmentation with a consortium that includes native microorganisms that will stimulate the adaptation of external microorganisms [77,78].

64, isolated three biosurfactant-producing strains, *Pseudomonas* sp. E39, E311, and E313 from hydrocarbon-contaminated soil, and evaluated their biosurfactant potential and diesel biodegradation capacity. The study reported a reduction in TPH of 70% in bioreactors that were inoculated with the control *R. erythropolis* T902.1. For the isolated strains E311, E39, and E313, a relatively low TPH removal rate (approximately 55%) was reached. The E311 strain displayed lower lipopeptide yields, but the highest biodegradation efficiency. This suggests that low biosurfactant production could be sufficient. High hydrocarbon solubility could have an inhibitory effect on microorganisms owing to the toxicity of the cell, as has also been reported in the literature dealing with biostimulation. A novel biosurfactant producer, *Serratia* sp. strain KDS, was studied by [49]. Its efficiency was evaluated using germination tests on contaminated soil samples during a pilot-scale bioremediation experiment. This bacterium reached a maximum percentage of biodegradation of 87.54% for diesel and 85.48% for kerosene. The germination rate and the highest height of the treated plants indicated that this microorganism produced a non-cytotoxic biosurfactant.

#### 4.1.3. Complementary strategies: biostimulation and bioaugmentation

According to [69], bioaugmentation and biostimulation are complementary strategies (see Table 2]. They found that using both strategies, oil degradation did not present significant differences (p > 0.05) in comparison with the control group with natural attenuation at 60 d (57.92% and 59.40% removal, respectively). [79], supported the hypothesis that biodegradation efficiency increases when the applied biosurfactant is produced by a native or isolated microorganism from a polluted site. They applied an isolated consortium jointly with sophorolipid biostimulation to treat petroleum-contaminated soil. They found that TPH biodegradation increased from 12.2% to 44.5% in the treatment that used only the isolated consortium, and to 57.7% in the treatment with the isolated consortium plus 1.5 g of sophorolipid per kg of dry soil. They concluded that the biosurfactant improved TPH desorption from the solid matrix to the biofilm, served as a carbon source, and promoted an active co-metabolism process. These results are supported by [15], who compared the two bioremediation strategies in their study. They found that for diesel treatment, the use of a consortium with biosurfactant-producing microorganisms achieved the best results. They reported an improvement in diesel biodegradation of 12.19% and 15.35% when SPB1 lipopeptide was used as biological emulsifier. Nevertheless, the best degradation rate (32.67%) was obtained with *B. subtilis* SPB1 and *Acinetobacter radioresistens* RI7.

In conclusion, the use of an isolated consortium that includes biosurfactant-producing strains showed better results for crude oil remediation because of their assistance in the availability of carbon sources for degrading microorganisms. Nevertheless, further research is required in this area.

### 4.2. Polycyclic aromatic hydrocarbons (PAHs) bioremediation

PAHs are hydrophobic organic compounds with complex chemical ring structures that are highly toxic and harmful to health [80]. This kind of pollutants is mainly found as a result of anthropogenic activities, such as the use of wood preservatives like creosote (which is composed of approximately 85% PAHs) or the burning of fossil fuels and other industrial activities [81]. Moreover, for high molecular weight hydrocarbons, bioavailability is reduced owing to their absorption and binding to organic solid matrices, thus complicating the use of bioremediation technologies. Therefore, biosurfactants or biosurfactant-producing bacteria are used to improve the bioavailability of these compounds [17,82,83]. The studies were conducted using samples from contaminated sites or soils spiked with contaminants to simulate the same bioremediation conditions. The reviewed cases of PAHs bioremediation using biosurfactants are summarized in Table 3.

**Table 3.** Reviewed cases for PAHs bioremediation with biosurfactant

Reported experiment	Biosurfactant	Microorganism or consortium	Substrate	Biosurfactant efficiency (%)	Reference
***Biostimulation***					
Research on the application of phenol in PAHs biosurfactant solution and how it improves the biodegrading of PAHs for *in-situ* application	Glycolipid	*Pseudomonas aeruginosa* S5	Sludge-adsorbed PAHs collected from aerobic bioreactors of coking wastewater treatment system	43.1% increase of PAHs bioavailability with biosurfactant and 49.2% with biosurfactant and phenol	7
Determination of rhamnolipid effect on PAHs solubilization and biodegradation in bioremediated contaminated soils by desorption	Rhamnolipid	-	*S1*: wood treatment plant soil mixed with agricultural sandy soil *S2*: 5 months biopiles soil with creosote *S3*: Several years biopiles soil	Significant result on S3, 50.7% pyrene mineralization as example of PAHs	85
Biosurfactant effect on the bioavailability and subsequent biodegradation of PAHs compounds	Lipopeptide	*Pseudomonas aeruginosa* CB1	Soil contaminated with creosote	86.5% PAHs degradation with biosurfactant	3
Produced biosurfactant effect on PAHs biodegradation by a microbial consortium from a previously bioremediated soil	Lipopeptide	*Bacillus cereus* SPL-4	Soil contaminated for more than 20 years	At 0.2 and 0.6% (w/w) lipopeptide: 51.2% – 64.1% of 4-ring 55.0% – 79.0% of 5- and 6- ring, PAHs removal respectively.	81a
Produced lipopeptide effect on pyrene degradation by a microbial consortium	Lipopeptide	*Pseudomonas viridiflava* and *Pseudomonas nitroreducens*	Liquid culture medium with pyrene 200 µL	At 600 mg L^−1^ and 300 mg L^−1^ biosurfactant concentration: 83.5% and 67.0% pyrene biodegradation, respectively	86
Extraction and isolation of biosurfactant producer bacterial populations from a PAHs polluted soil and method developing for biosurfactant production and recovery	Biosurfactant extract	Mainly *Enterobacteriaceae* and *Pseudomonas*	Soil and water samples of a plume area contaminated with PAHs, BTEX and other hydrocarbons of a former coke plant	Solubilization ratios of 0.21 mg g^−1^ for phenanthrene, 0.12 mg g^−1^ for pyrene and 0.01 mg g^−1^ benzo[α]pyrene	53
***Bioaugmentation***					
Isolated biosurfactant producer microorganism capacity for solubilizing PAHs and its application on *in-situ* remediation	Glycolipid	*Pseudomonas aeruginosa* S5	Standard PAHs solution	PAHs removal of: 27.1% by the co-metabolic group (native microorganisms plus glucose) 61.5% with the co-inoculation of S5 strain	87
Evaluation of PAHs mineralization with Brij-35 and rhamnolipid surfactants on soil native microorganisms and on a bioaugmentation group	Rhamnolipid	*Mycobacterium vanbaalenii* PYR-1	Clay soil, and sandy soil contaminated with pyrene	Results based on sequencing and phylogenetic investigation of soil communities	84
Identification of a biosurfactant producer bacteria during PAHs degradation and biosurfactant characterization	Mono- and di-rhamnolipid	*Bacillus algicola* 003-Phe1, *Rhodococcus soli* 102-Na5, *Isoptericola chiayiensis* 103-Na4, and *Pseudoalteromonas agarivorans* SDRB-Py1	Marine sediments polluted with crude oil	>85.0% crude oil degradation by the consortium and 48.0–72.0% of crude oil desorbed by the produced biosurfactant extract	88

#### 4.2.1 Biostimulation for PAHs bioremediation with biosurfactants

Biostimulation and bioaugmentation are the most used strategies. For biostimulation, biosurfactants are added to increase PAHs bioavailability in the native microbiome. The efficiency reported for biostimulation strategies ranged 43.1–86.5% (Table 3), with glycolipids as the most frequently used biosurfactant.

84,reported changes in the bacterial soil community and analyzed PAHs mineralization using the chemical surfactants Brij-35 and rhamnolipids. The results showed no significant effect on the Shannon index when Brij-35, rhamnolipid, or both were used. Only a high dose of rhamnolipid (1400 µg g^−1^) caused an index decrease owing to its antibacterial activity. When rhamnolipids were used, the most dominant microbial genus present in clay soil analyzed by [84], was *Mycoplana* (67%). It uses rhamnolipids as a carbon source, causing a decrease in the abundance of known PAHs degrading microorganisms, such as *Bacillus, Mycobacterium, Sphingomonas, Rhodococcus* and *Kaistobacter* present in other soils. However, *Bacillus* was the predominant genus in the sandy soil (58%), where the highest dose of rhamnolipid was applied, starting pyrene mineralization after 50 days. This result shows that the biosurfactant was used as a preferential carbon source by microbes, as reported by [85]. These authors also argued that the desorption of PAHs in non-treated soils occurs slowly, because soil microorganisms may use rhamnolipids as the main carbon source before pyrene. In contrast, in previously bioremediated soils where fast-desorption PAHs were removed, rhamnolipids at concentrations above their CMC optimized the bio-accessibility of recalcitrant PAHs (slow-desorbing PAHs) for degrading microorganisms.

When higher doses of biosurfactants are applied, pollutant degradation is limited or soil microbial diversity decreases drastically, making the process inefficient. Several authors have reported an inhibitory effect on cell growth when high doses of biosurfactant are applied as a result of biosurfactant overdose, the accumulation of inhibitory molecules from incomplete metabolism, and stress response caused by the high solubilization of the pollutant, which can interfere with cell membrane permeability [24,86]. Studies conducted by [86], in which three lipopeptide concentrations were tested, support this hypothesis. Using 300 mg L^−1^ and 600 mg L^−1^ lipopeptides eliminated 68% and 83% of pyrene, respectively. However, at the highest dose (900 mg L^−1^), the degradation of pyrene was reduced to 57% owing to the inhibition of autochthonous bacteria.

81], concluded that the removal rate of high-molecular-weight PAHs was significant when a bacterial consortium from a previously bioremediated soil was supplemented with a lipopeptide biosurfactant produced by *Bacillus cereus* SPL-4. At a concentration of 0.2% and 0.6% (w/w) lipopeptide, it removed PAHs of 4-rings (51.2% and 64.1%, respectively), and 5- and 6-rings (55% and 79%, respectively) in comparison with the free-biosurfactant control. These results indicate that biosurfactants accelerated the degradation kinetics of 5- and 6-rings PAHs at a dosage of 0.6% (w/w), with a total removal of 79.9%. According to [3], the presence of biosurfactants in bioremediation processes increases the degradation of PAHs between 27% and 86.5%, increasing their concentration in the aqueous phase for a rapid biodegradation. However, in this case, co-metabolism enzymes were used to support the biodegradation process of low-molecular-weight PAHs.

3], also studied the effects of nutrients and lipopeptides (3 g kg^−1^) addition for naphthalene and phenanthrene degradation. At 45 d, in the reactor supplemented with biosurfactant, the degradation was 74% for naphthalene and 88% for phenanthrene. Moreover, in the reactor supplemented with the biosurfactant plus NH_4_NO_3_ and KH_2_PO_4_ as nutrients, the biodegradation of naphthalene was 51% and 81%, respectively, for phenanthrene in comparison with the biotic control (30% and 59% degradation, respectively). These findings confirm that the biodegradation of persistent PAHs can be enhanced by biostimulation with the biosurfactant alone or with the simultaneous use of nutrients (N and P). Nevertheless, nutrients can enhance biosurfactant efficiency. It has been observed that low molecular weight organic compounds can also promote the bioavailability of some pollutants. Amphiphilic compounds as phenol can work as a ‘pseudo co-surfactant’ decreasing the CMC of the biosurfactant and forming a co-matrix with the carbon source. Consequently, a mixed micelle with the biosurfactant aids PAHs dissolution and boosts bioremediation. This was observed by [7], who improved PAHs solubilization from 27.7% to 43.1% using a microbial biosurfactant extract produced by *P. aeruginosa* strain S5 and increased it to 49.2% with the addition of phenol. However, the efficiency values should present significant differences because nutrient supplementation is reflected in the cost of the process.

#### 4.2.2. Bioaugmentation for PAHs bioremediation with biosurfactants

Bioaugmentation is a strategy that has been reported by many authors as a potential alternative in contaminated environments, with a biodegradation efficiency from 27.12% to more than 85% (Table 3). [87], simulated the conditions of a contaminated environment using a PAHs solution to evaluate the biodegradation capacity of a wastewater isolate strain with potential biosurfactant production. The removal efficiency of total PAHs was improved from 17.14% to 27.12% in comparison with the control group. However, the highest degradation rate was found in the bioaugmentation group with the inoculation of the *P. aeruginosa* S5 strain (PAHs biodegradation was 61.47%). [24], found that with the supplementation of 2.5 mg L^−1^ MELs biosurfactant produced by *Pseudozyma* sp. NII 08165, the microorganism *Pseudomonas putida* achieved a maximum hydrocarbon biodegradation of 46%. Moreover, *Pseudozyma* sp. NII 08165 alone can also degrade kerosene and diesel, which are potential genera for bioremediation.

Several authors have confirmed that biosurfactant-producing bacteria can take advantage of a microbiological consortium and metabolize diverse classes of hydrocarbons with *P. aeruginosa* being the most used [6, 17, 52, 84]. [88], isolated an autochthonous biosurfactant-producing bacterial consortium from polluted samples of marine sediments to evaluate its PAHs degradation capacity. It is composed of *Bacillus algicola* 003-Phe1, *Rhodococcus soli* 102-Na5, *Isoptericola chiayiensis* 103-Na4, and *Pseudoalteromonas agarivorans* SDRB-Py1, which commonly produces monorhamnolipid and dirhamnolipid biosurfactants. These results are in accordance with those of [53], who described that PAHs bioavailability increased when the soil consortium was composed of the genera *Pseudomonas, Enterobacteriaceae, Microbacterium* and *Rhodanobacteraceae*.

Based on these results, a promising research area should focus on the isolation of hydrocarbon-degrading biosurfactant-producing microorganisms. Thus, the use of autochthonous microorganisms that have both capacities (PAHs degradation and biosurfactant production) accelerates the biodegradation process.

### 4.3. Bioremediation of metal-contaminated environments with biosurfactants

Metals in soils and water environments are hazardous to humans and other living organisms because of their bioaccumulative characteristics. In addition, heavy metals such as cadmium, copper, arsenic, chromium, mercury, and lead persist in the environment [89]. Metals are not biodegradable; however, their toxicity and mobility can be modified by changing their chemical state (using alkylation or redox processes). Microorganisms are essential parts of the process of accumulating or using them in electron transfer reactions. The remediation of a solid matrix depends on variables such as the particle size of the material, pH, metal exchange capacity, metal persistence and contamination time, which vary from site to site [18,90].

Two technologies have been developed to remediate contaminated habitats. The first involves immobilizing the metal on a matrix that is strongly bound to the soil to minimize metal migration. However, this is not a definitive solution and long-term monitoring is required. The second technique is known as soil-washing technology. This promotes the mobility of the metal and its migration to the liquid phase through desorption and solubilization. This is considered a permanent solution because it allows the reuse of remediated soil [5,91]. A disadvantage of these washing technologies is the use of acids or chelating agents, such as ethylenediaminetetraacetic acid (EDTA), which reduces the fertility of the soil and alters its physicochemical properties owing to the dissolution of minerals. Furthermore, the use of EDTA is not highly recommended from a health and safety perspective because of its low degradation rate and the formation of a metal-EDTA complex [19].

18], suggested that biosurfactants decrease heavy metal toxicity and promote microbial activity in soil. Apart from their effect on the metabolic activity of microorganisms, biosurfactants facilitated the solubilization, dispersion, and sorption of metals and allowed the reuse of the treated soils. Metal treatment can be carried out by *in-situ* methods (phytoremediation, chemical mobilization, electrokinetic extraction, soil flushing, and surface capping) or *ex-situ* methods (soil washing and solidification); soil flushing and soil washing are the most recommended methods for biosurfactant-producing microorganisms [19]. The metal removal efficiency depends on the structure and properties of the biosurfactant-metal interactions. For example, it has been reported that at two-fold CMC, the biosurfactant produced by *Bacillus sp*. shows high heavy metal removal, mainly for Cd (99.93%), Pb (97.73%), Mn (89.5%), and Hg (75.5%) showing the formation of a co-precipitate [92]. Biosurfactant concentration has been reported to be an important parameter. It has been demonstrated that removal efficiency gradually increases with concentration [93].

The effects of biosurfactants on soil quality must also be considered for a complete evaluation of biosurfactants in these contaminated environments. Accordingly, plant germination and growth tests are commonly used in treated soils. However, these studies focused on biosurfactant capacity for metal removal, and the quality of the remediated soil has not always been evaluated (Table 4).

**Table 4.** Summary of reviewed cases polluted with metals

Reported experiment	Biosurfactant	Microorganism or consortium	Substrate	Pollutant	Biosurfactant efficiency [%]	Reference
***Metals Removal***						
Evaluate the efficiency of a biosurfactant extract on soil contaminated with heavy metals	Biosurfactant extract	*Candida sphaerica* UCP0995	Soil samples from an automotive battery industrial	Zn Fe Pb	Removal rates for Zn, Fe and Pb: 90%, 95%, 79% by biosurfactant extract. 65%, 75%, 57% by 0.1% biosurfactant 68%, 80% and 65% by 0.25% biosurfactant 87%, 89% and 70% by 2.5% biosurfactant solution, respectively.	94
Sludge metal decontamination by electroremediation treatment with rhamnolipids and glutamic acid (GLDA) as electrolytes	Rhamnolipid	-	Wastewater sludge with concentrations of heavy metals	Cu Zn Cr Pb Ni Mn	Cu, Zn, Cr, Pb, Ni and Mn removal: 65%,57%,49%,47%,60% and 70% by biosurfactant 71%, 82%, 89%, 60%, 88% and 70% by GLDA plus biosurfactant, respectively.	95
Evaluate the application of a produced biosurfactant on sand decontamination and an aqueous effluent containing heavy metals	Biosurfactant extract	*Candida tropicalis*	Sand artificially contaminated with a metallic solution	Zn Cu Pb	Removal rate for Zn, Cu and Pb: 30, 80%, 15% by purified biosurfactant 60%, 55% and 10% by crude biosurfactant, respectively	5
***Toxicity test***						
Evaluate the growth of two species of crops, *Triticum aestivum* and *Capsicum annum* at different pollutants concentrations (10 ppm and 20ppm each) under biosurfactant stimulation	Lipopeptide	*Brevibacillus brevis* BAB-6437	Biosurfactant supplementation 3% (w/v)	Cr Azulene	Germination percentages of wheat crops and pepper crops: At 20 ppm Cr: 58%, 54.5% and At 20 ppm azulene: 58%, 59%, respectively.	2
Evaluate the effect of biosurfactant by larvicidal activity *Anopheles culicifacies* and toxicity test on onion bulbs *Allium cepa* germination	Cyclic lipopeptide (CL)	*Bacillus tequilensis* CH	Larvicidal effect: 85, 100, 110, 130 and 145 ug mL^−1^ biosurfactant Toxic effects: 1ppm Cd + 0.1 mg mL^−1^ CL, 0.1 mg mL^−1-^CL	CdCl_2_	*Larvicidal effect*: 50% of mortality at biosurfactant concentration of 110 ug mL^−1^ *Toxicity effect*: at 1ppm Cd+0,1 mg mL^−1^ CL 52%, 8% and 36% with 0.1 mg mL^−1^ CL 76%, 44% and 20% of normal division, abnormal division, and non-dividing cells, respectively	97
Evaluate the biosurfactant effect on the growth of heavy metal accumulating plants *Bidens pilosa*. and *Medicago sativa*	Sophorolipid (SL)	-	Growth effect: seedlings with 10 mL of SL at 0.5% Assisted phytoremediation: SL and Cd at 1.9 g pot^−1^ and 29.2 mgkg^−1^, respectively	Cd(NO_3_)_2_.4H_2_O	*Growth effect*: Shoot heights of *B. Pilosa* and *M. sativa* under SL treatment were ~11% and 16.85% respectively, more than the untreated group Biosurfactant assisted phytoremediation: In *B. pilosa* SL augmentation decreased proline concentration (18.2 µmoles g^−1^)	96

The most used biosurfactants in these bioremediation processes are molecules with electric charge. Biosurfactant binding capacity permits the formation of a stronger complex between the anionic or cationic biosurfactant and metal ions (ionic bonds) than the complex soil metal. This favors their desorption from the soil matrix [82]. Consequently, anionic biosurfactants are more commonly used for metal removal than cationic ones. [94], used an anionic biosurfactant extract from *C. sphaerica* and achieved the highest removal rates of 79%, 90%, and 95% for Pb, Zn, and Fe, respectively. They showed that the biosurfactant had a higher affinity for metal cations (Fe and Zn).

5,and [95], applied biosurfactants in combination with different agents that affect metal removal. [5], showed that the anionic biosurfactant produced by *Candida tropicalis* had no specific affinity for the metals analyzed when combined with additives such as HCl and NaOH. However, biosurfactant alone was efficient in the removal of Zn and Cu, with elimination rates between 35% and 80%, while the highest Pb elimination was 15%. In the application of the cell-free broth containing biosurfactant extract on sand columns, 60%, 55% and 10% of Cu, Zn, and Pb were removed from the sand, respectively. Thus, this study concluded that the biosurfactant could be used in the treatment of soil polluted with heavy metals, considering the interaction with additives, concentration, and soil characteristics. [95], used rhamnolipids and reported removal efficiencies for Cu, Zn, Cr, Pb, Ni and Mn of 64.8%, 56.8%, 49.4%, 46.6%, 60.4% and 69.6%, respectively. However, the best results were obtained with the simultaneous application of GLDA (N,N-dicarboxymethyl glutamic acid tetrasodium salt, a strong chelating agent) and rhamnolipids with removal efficiencies for Cu, Zn, Cr, Pb, Ni and Mn of 70.6%, 82.2%, 89%, 60%, 88.4% and 70%, respectively. In conclusion, this biosurfactant can remove organic matter that prevents the chelating action of GLDA, which is used to improve remediation activities in electrokinetic treatment.

In the study by [19], rhamnolipids were used and Cd removal efficiency of 85% at pH 7 was reported. These results provide further support for the use of rhamnolipids for metal bioremediation as a well-characterized anionic biosurfactant, which has high affinity for toxic metals such as cadmium. Moreover, rhamnolipids can form micellar and lipid aggregates or lamella-like structures despite the pH of the remediated soil [18].

#### 4.3.1 Biosurfactants and phytoremediation

Another way to remove metals in soils is phytoremediation. However, it must be considered that plants accumulate metals and cannot absorb them completely, a problem that can have repercussions in biomagnification through the food chain [95].

Nevertheless, in phytoremediation, biosurfactants are used to support plant growth and improve soil quality. In this case, plants contaminated with metals are treated, and the application of biosurfactants favors their development either in the germination phase or in correct cell division. [2], evaluated the effects of a lipopeptide produced by *Brevibacillus brevis* BAB-6437 on plants contaminated with two metal textile pollutants. Germination assays of wheat and pepper crops concluded that the application of biosurfactant increased the weight of leaves in plants contaminated with azulene and chromium at a concentration of 20 ppm. Moreover, a decline in the proline concentration (a sign of oxidative stress in plants) was observed as a positive effect of biosurfactants supplementation. Shah and Daverey [96] reported sophorolipid augmentation in Cd-contaminated soils. A decrease in proline concentrations was found in a metal-accumulating plant, *Bidens pilosa* (18.2 µ moles proline g^−1^ and 40.2 µ moles proline g^−1^ in the treated soil and control group, respectively), and Cd toxic effects were reduced significantly. In addition, sophorolipid improved *Medicago sativa* and *B. pilosa* shoot and root growth and enhanced plant root permeability, consequently increasing nutrient uptake and phytobiomass. The reviewed cases support the fact that biosurfactant augmentation in phytoremediation cases can reduce pollutant toxicity and promote soil respiration and nutrient uptake, enhancing microbial activity and plant growth [18].

The effects of a cyclic lipopeptide and Cd on plant germination were also examined by [97]. Results showed that in onion germination assays, in the absence of Cd ions, 60% of the cells were in the division phase with less than 4% of any visible abnormality. However, in the presence of 1 ppm Cd, most of the root cells were not in any phase of mitosis (56%), and approximately 40% of the cells showed abnormal mitotic division. When 1 ppm of Cd was combined with 0.1 mg mL^−1^ of lipopeptide, a less adverse effect of Cd was observed as 52% of the cell division appeared normal, with only 8% of abnormal division. Without Cd and at a dose of 0.1 mg mL^−1^ of lipopeptide, the dividing cells showed no adverse effects. These results suggest that Cd has a profound mutagenic effect on cell division, which may be related to the occurrence of abnormal metaphase, anaphase, and nucleus elongation in plant cells. Nevertheless, the presence of lipopeptides can reduce this adverse effect without causing toxic effects on the onion cells.

Biosurfactants can play an important role in the creation of complex biosurfactant-metals by different attraction or repulsion forces owing to their binding capacity. This suggests that they can be used instead of persistent chemical chelating agents. In addition, biosurfactant augmentation in phytoremediation must be considered to reduce metal toxicity and improve plant growth. A research field on plant metabolic pathways to explain how they use biosurfactants and how they protect plants under stress conditions is open.

### 4.4. Biosurfactants as biocidal agents

Biosurfactants at relatively high doses can reduce microbial biodiversity. Therefore, they can also be used as biocidal agents. Several studies have claimed that the bactericidal activity of antimicrobial agents (biocides) contributes to the generation of free hydroxyl radicals, cell disruption, and bacterial death. Biosurfactants, at concentrations above their CMC, often alter microorganisms’ metabolic pathways and cell surface properties, causing cell disruption [24].

All literature studies on this topic aim to reduce the proliferation of organisms that can have negative effects on human activity (Table 5). Some authors, such as [22, 35, and 98], have exposed different microorganism strains at different biosurfactant concentrations to evaluate their effect on microbial growth. [97], analyzed the effect of biosurfactants on *Anopheles culicifacies*, the transmission vectors of malaria.

**Table 5.** Biosurfactants biocidal activity evaluation against

Reported experiment	Biosurfactant	Affected microorganism	Remarkable/ Biosurfactant efficiency (%)	Reference
Test of antibacterial activity of the biosurfactants produced by *Candida albicans* (CA-B) and *Candida glabrata* (CG-B)	Sophorolipid	*Escherichia coli* MTCC 723 and *Bacillus subtilis* MTCC 441	Best results at biosurfactant concentration of 60 mg L^−1^: CG-B biosurfactant killed 65.8% of *B. subtilis population* and CA-B extract a 24.2%	35
Evaluation of biosurfactant biocide activity against phytopathogens	Rhamnolipid	*Sclerotium rolfsii, Fusarium oxysporum, Phytophthora nicotianae and Macrophomina faseolina*	Best results at several tested biosurfactant concentrations: At 300 µg mL^−1^, 60.46% against *M. phaseolin*a At 400 µg mL^−1^, 55% against *F. oxysporum* At 450 µg mL^−1^, 64% for against *P. nicotianae*	98
Determine biosurfactant effectiveness on potato leaves against zoospores of phytopathogens	Rhamnolipid	*Phytophthora infestans*	At 0.2% biosurfactant concentration growth inhibition of *P. Infestans* and no-phytotoxicity	22
Biosurfactant production, characterization, and antibacterial evaluation by half maximal inhibitory concentration (IC50) and minimum inhibitory concentration (MIC) for a prospective environmental application	Rhamnolipid	*Enterococcus hirae and Escherichia coli*	IC50 concentrations were estimated as: 87 μg mL^−1^ for *E. hirae* 106 μg mL^−1^ for *E. coli* MIC value was obtained as: 100 μg mL^−1^ for *E. hirae* 150 μg mL^−1^ for *E. coli.*	99

In these studies, the results were expressed as the percentage of mortality of the different analyzed life forms. According to [35], sophorolipids produced by *Candida albicans* (CA-B) and *Candida glabrata* (CG-B) show high antibacterial activity against gram-positive microorganisms. The elimination rate for the sophorolipid CG-B was 65% of the total population of *B. subtilis* strain MTCC 441, and 24.2% for the sophorolipid CA-B. Furthermore, negligible effects on *Escherichia coli* MTCC723 of 3.6% and 4.4%, respectively, were observed for both sophorolipids. According to [99], rhamnolipids present a minimum inhibitory concentration ranging 50–1600 µg mL^−1^. Higher biosurfactant concentrations are needed against gram-negative bacteria because of their trick lipopolysaccharide outer membrane. MELs also have antibacterial activity against gram-positive microorganisms, and several studies have reported that some gram-negative strains (*Pseudomonas* genus) are more sensitive to MEL biosurfactants [24].

22], evaluated the effects of rhamnolipids on potato pests caused by *Phytophthora infestans*. Pathogenic bacterium removal was expressed in the affected area of the leaf from the treated potato compared with the control group, which had an area of 9.8 cm^2^. Leaves treated with higher doses of rhamnolipids showed an affected area of 0.06 cm^2^ compared with the control on the fifth day of inoculation. However, at lower concentrations (0.15% and below), an affected area of 1.5 cm^2^ was observed, which increased as the dose of rhamnolipid was reduced by leaf application. Notably, a slight phytotoxicity effect was observed at the highest concentration of biosurfactant (0.3%). The rhamnolipid effect indicates that zoospores are likely lysed by the presence of the biosurfactant. The high sensitivity of *P. infestans* zoospores to biosurfactants suggests that they can be used to prevent late spread on potatoes once an infection has been detected [22,98].

98], evaluated the use of rhamnolipids as biocides for phytopathogens. The inhibition produced by rhamnolipids in the mycelial growth was 60.46% at 300 µg mL^−1^, 55% at 400 µg ml^−1^ and 63.63% at 450 µg mL^−1^ against *Macrophomina phaseolina, Fusarium oxysporum* and *Phytophthora nicotianae*, respectively. There were no significant differences between the antifungal activities of rhamnolipids produced using mango kernel oil and mango kernel oil plus glucose. Nevertheless, the substrate influenced the rhamnolipid mixture which was composed of 53.71% hydrophilic congeners when the medium was supplemented with glucose, and 35.08% when mango kernel oil was used alone. According to [100], rhamnolipids mixture and purified mono- and di-rhamnolipids congeners at concentrations of 45–1500 mg L^−1^ decrease the growth of *Aspergillus flavus* without significant difference. However, when rhamnolipids mixture is used, aflatoxins production is significantly inhibited in a range of 93.9–99.5% owing to the synergistic effect of the congeners and the stress condition they induce.

These results suggest that the antifungal properties of rhamnolipids produced by *Pseudomonas sp*. showed an inhibitory effect against several fungi and phytopathogens, and that there was no relationship between production substrates and biosurfactant properties. However, there was a relationship between the substrate and the proportion of congeners obtained.

Another biocidal effect of lipopeptide biosurfactants on the larvae of *Anopheles culicifacies* was reported by [97]. According to the obtained results, the LC50 value for the dose of cyclic lipopeptide was 110 μg mL^−1^ in two days, and death increased to 60% at higher doses. In this case, the biocidal effect is caused by the reduction in water surface tension. Mosquitoes lay their eggs (which later become larvae) on the surface of the water to obtain oxygen from the air. Therefore, they keep their bodies parallel to the water surface to breathe. However, owing to the addition of a lipopeptide, the surface tension of water decreases. Therefore, the eggs cannot adhere to the surface of the water, and the larvae sink to the bottom, causing death by suffocation.

101], also argued that in any case, the results suggest that another relevant application of surfactants may be to mitigate the spread of pathogens that affect human activity. This function has traditionally been achieved through the application of chemical pesticides. The literature shows that biosurfactants have a wide range of applications that depend on their properties and concentrations. For example, some authors have reported that sophorolipids have antiviral activity against RNA viruses, suggesting their possible use against SARS-CoV-2 [22]. Their effect on the organisms or media to be treated should be studied in advance to determine the optimum concentration according to their application.

### 4.5. Other environmental applications

As biosurfactants have applications other than those described above, some biosurfactant selection criteria are detailed for their application in the environmental field (Table 6). Recent studies have shown that biosurfactants can be used in agriculture. [32], summarized the agricultural applications of MSAC and concluded that biosurfactants promote plant growth through phytohormone production, microbial stimulation, and an increase in nutrient availability in soil. For example, *Bacillus* sp. J119 biosurfactant promoted Sudan grass, tomato, and canola maize growth. Furthermore, the addition of biosurfactants improves plant resistance to biotic and abiotic stresses by acting as biocontrol agents.

**Table 6.** Selection criteria for biosurfactant application on an environmental field

Biosurfactant environmental application criteria
1. Pollutant properties and characteristics
2. Biosurfactant properties mainly critical micellar concentration (due to its biocidal activity)
3. Biosurfactant use characteristic: solubilization, mobilization, emulsification, etc.
4. Biosurfactant stability at environmental work conditions
5. Process development *in-situ* or *ex-situ*
6. Competitive producer microorganisms or consortium
7. Nutrients or precursors request
8. Environmental Interest *Bioremediation* *Biostimulation and/or bioaugmentation* *Metal immobilization or soil washing* *Phytoremediation* *Agricultural use* *Solvents removal* *Biocidal application*
9. Post-essay analysis

Atrazine is a well-known herbicide which has effects that serve as clear examples of the negative environmental consequences induced by chemical compounds. [21] obtained surfactin lipopeptides from *Bacillus velezensis* MHNK1 and confirmed that at different concentrations, the biosurfactant can improve the biodegradation of atrazine in agricultural soil. Results showed that the degradation rate of atrazine after 2 days of incubation of the strain without surfactin was only 28.24%. When combined with the different doses of surfactin based on the CMC (equal to CMC, two-fold the CMC, and three-fold the CMC), the rate was 21.54%, 31.33%, and 18.03%, respectively. When the optimal surfactin CMC dose (two-fold) was applied, bacterial growth was enhanced and atrazine biodegradation was improved by increasing its solubility and bioavailability. However, at three-fold the biosurfactant CMC, atrazine biodegradation decreased, probably because of an antibacterial effect against degrading microorganisms.

In a study by [102], the effect of produced rhamnolipids on improving the solubilization of persistent chlorinated pesticides (endosulfan ES, α and β isomers and hexachlorocyclohexane HCH, γ-isomer) was evaluated. Five different concentrations (45 mg L^−1^, 60 mg L^−1^, 75 mg L^−1^, 90 mg L^−1^ and 105 mg L^−1^) of each surfactant were added (rhamnolipid-produced IITR51, commercial rhamnolipid JBR425, and synthetic surfactant Triton X100). They found that the addition of the produced rhamnolipid improved the solubility 7.2 times for α-endosulfan, 2.9 times for β-endosulfan and 1.8 times for γ-CH, compared with their respective controls. In addition, higher solubilization was observed for α-endosulfan and β-endosulfan for rhamnolipid IITR51 compared with commercial rhamnolipid JBR425 and the synthetic surfactant Triton X100. [103], described that biosurfactants can also be used to remove pesticides from contaminated environments, making them accessible for microbial enzymatic breakdown. They proposed the use of microbial metagenomics to detect new biosurfactant-producing species, metabolic pathways, and genes involved in novel biosurfactant discoveries, such as N-acyl amino acids and palmitoyl putrescine.

104], evaluated the effects of monorhamnolipid biosurfactants on a sieved soil that was manually contaminated with two explosives (trinitrotoluene (TNT) and pentaerythritol tetranitrate (PETN)) to analyze the improvement of natural soil attenuation in a laboratory-scale bioreactor. A biosurfactant was added at a concentration of 120 mg L^−1^ to evaluate its effect on the degradation rates. After 90 days, TNT and PETN were removed with biodegradation efficiencies of 92.5% and 52%, respectively, indicating that the use of biosurfactants overcomes the biodegradation of nitroaromatic compounds.

The biosurfactants produced by *Corynebacterium aquaticum* and *Corynebacterium* spp. CCT-1968, using fish waste, sugarcane bagasse (SBC), glycerol, or petroleum sludge as a carbon source, was evaluated for its application in paint removal, a novel contribution described by [44]. However, these microorganisms are not often used for biosurfactant production. Nonetheless, they can produce biosurfactants with effective emulsifying properties. The best results were obtained in the fermentation process with *C. aquaticum* using 3% of fish waste and 3% SCB. *Corynebacterium* spp. CCT-1968 using SCB as carbon source presented a high emulsifying activity (88.3%); however, surface tension did not show a large decrease. Results with glycerol and petroleum sludge demonstrated that they are not good carbon sources for biosurfactant production using this microorganism. Nonetheless, *C. aquaticum* can produce high-quality biosurfactants at high concentrations of petroleum waste. This study confirms the fact that many studies have reported that emulsifying activity and surface tension are not necessarily related; one biosurfactant can be a good agent for reducing the surface tension and have a low emulsifying capacity. In addition, this type of biosurfactant shows promising solubilization capacity, which can avoid the use of toxic solvents for paint removal.

## 5. Limitations and prospects of biosurfactants in environmental applications

Biosurfactant production efforts have been focused on the use of non-food competitive feedstocks, in the revalorization of industrial and organic waste, and in the use of new bioprocessing strategies such as solid-state fermentation. Nevertheless, a crucial step for future industrial biosurfactant production is the comparison of SmF and SSF using the same substrates to evaluate the productivity and future commercially viable operations. Some authors have reported a biosurfactant crude extract without downstream processing as the final fermentation product, which is a crucial step in achieving acceptable product quality [58]. Novel purification techniques such as the use of nanoparticles and integrated gravity can be applied in the future. Their industrial approach is considered as an open research field [56,61].

From a macro point of view, biosurfactants for environmental applications have several advantages that allow their use under extremely polluted conditions. Based on the literature, there are three ways to use biosurfactants: biosurfactant crude extract, pure biosurfactant, or biosurfactant-producing microorganisms. In this regard, a comparison between the three application methods must be considered in future research. Additionally, considerable attention must be given when high biosurfactant concentrations are used because of their biocidal activity and possible enzymatic inhibition, which delays the remediation process and decreases microbial diversity. One option to overcome this problem is to isolate native biosurfactant-producing microorganisms from contaminated environments and find novel biosurfactants with the support of molecular tools such as microbial metagenomics.

From the reviewed literature, future research efforts should focus on biosurfactant support during the phytoremediation process and the isolation of microorganisms with both capacities: biosurfactant production, pollutant biodegradation, and their metabolic pathway interactions. Therefore, *in-situ* biosurfactant production can be used to decrease biosurfactant production and bioremediation costs.

## 6. Conclusions

Biosurfactants are the main microbial surface-active compounds used in environmental applications. They can improve soil fertility, protect plants under stress conditions, and act as natural biocides. However, their main environmental use is during bioremediation processes through biostimulation and/or bioaugmentation strategies making them promising alternatives for treating pollutants such as hydrocarbons, PAHs, and heavy metals. Potential biosurfactant application areas are being explored in agriculture for pesticide removal and phytoremediation processes. In comparison with synthetic surfactants, biosurfactants have several advantages such as low toxicity, stability under several conditions of temperature, pH, and salinity, and high biodegradability, which are among the most important characteristics for their applicability in this field.
